# Association of Lifestyle Behaviors with Quality of Life in Patients with COPD: A Cross-Sectional Study in Primary Care

**DOI:** 10.3390/jcm13164793

**Published:** 2024-08-14

**Authors:** Izolde Bouloukaki, Antonios Christodoulakis, Katerina Margetaki, Ioanna Tsiligianni

**Affiliations:** 1 Health Planning Unit, Department of Social Medicine, School of Medicine, University of Crete, 71500 Heraklion, Greece; izolthi@gmail.com (I.B.); katmargetaki@hotmail.com (K.M.); i.tsiligianni@uoc.gr (I.T.); 2 Department of Nursing, School of Health Sciences, Hellenic Mediterranean University, 71410 Heraklion, Greece

**Keywords:** COPD, healthy, behaviors, Greece, smoking, physical activity, alcohol consumption, BMI, primary care

## Abstract

**Background/Objectives**: The association between healthy lifestyle behaviors and their effect on quality of life among patients with COPD remains unclear. Therefore, the aim of this study was to explore the lifestyle behaviors and their association with the disease-specific quality of life among a primary care population with COPD in Greece. **Methods**: This cross-sectional study included 236 participants aged 40 years and older from the COCARE COPD study. The healthy lifestyle index (HLI) was created based on smoking, alcohol consumption, BMI, physical activity, and sleep duration, with each factor categorized as either healthy (1) or unhealthy (0). The HLI ranged from 0 (least healthy) to 5 (healthiest). COPD-specific quality of life was assessed using the COPD assessment test (CAT), where higher scores indicate poorer health status. Multiple logistic regression was used to analyze the association between HLI and CAT scores, adjusting for confounders. **Results**: Half of the participants were non-smokers or former smokers, while 92% reported consuming low levels of alcohol (less than 14 units per week). Additionally, 56% had a BMI below 30, indicating they were not obese. Surprisingly, only 32% engaged in regular exercise, with at least 150 min per week, and only 25% reported getting adequate sleep, ranging from 7 to 9 h per night. Importantly, poorer health status was inversely associated with non/former smoking (OR: 0.543, 95% CI: 0.282–1.049), physical activity (OR: 0.238, 95% CI: 0.122–0.463), and adequate sleep (OR: 0.337, 95% CI: 0.160–0.710). Patients with higher HLI scores were less likely to have poor health status. **Conclusions**: In conclusion, our findings indicate that a significant proportion of patients with COPD failed to adhere to a minimum of three out of five healthy behaviors. Additionally, a higher number of healthy lifestyle factors defined by a high HLI score were independently associated with a better disease-specific quality of life. This is particularly important for COPD where quality of life is in the epicenter of management. Therefore, healthcare providers could significantly improve the management of COPD and patient outcomes by targeting and improving these lifestyle behaviors with targeted and holistic intervention strategies.

## 1. Introduction

Chronic obstructive pulmonary disease (COPD) is widely acknowledged as one of the most prevalent non communicable diseases and is ranked as the third primary cause of mortality worldwide [1]. Characterized by progressive respiratory impairment, COPD, as it progresses, is frequently accompanied by several other comorbid symptoms and conditions, such as muscle wasting, bone loss, cardiovascular disease, anxiety, depression, and cognitive decline [2,3,4]. These manifestations can have a negative effect on the individual’s quality of life. Despite decades of dedicated research aimed at unraveling the causes and finding effective treatments for COPD, and the limitations of the current definition in capturing the fundamental nature of COPD, the medical community has been unable to achieve the same level of success in reducing the disease’s impact on health and mortality as it has for other major noncommunicable diseases [5,6]. Several evidence-based management approaches have been developed; however, the impact of COPD exacerbations and symptoms remains [7]. Consequently, there is an urgent need for innovative approaches to COPD management [8,9,10]. One such approach is “Treatable traits” (TTs), which is a precision medicine strategy for chronic airway diseases [9]. The TTs approach involves a thorough evaluation of every patient to identify specific characteristics and implement a tailored for each patient approach to their care [8]. Traits are categorized into three domains, namely, pulmonary, extra-pulmonary, and behavioral/lifestyle risk factors [8].

The implementation of comprehensive TTs models of care has demonstrated effectiveness in improving patient outcomes for patients with COPD [11,12]. However, many TTs models focus on pulmonary traits and overlook traits related to behavioral and lifestyle risk factors. Furthermore, most of the research has been conducted in tertiary care facilities, while the available evidence indicates that there are notable differences in the prevalence of the TTs between primary care and tertiary care [12]. In light of the above considerations and the necessity for more holistic healthcare models, it is crucial for new models to extend their focus beyond pulmonary traits and give priority to behavioral and lifestyle risk factors that are controllable, common, and are associated with COPD health status [13]. This is especially important in primary care settings, as it is within these settings that primary care physicians can best recognize and advise lifestyle modifications [14].

The World Health Organization (WHO) targets four major lifestyle risk factors that can be modified to reduce the burden of chronic diseases [15]. These include physical inactivity, unhealthy diet, high alcohol consumption, and smoking. Furthermore, there is substantial evidence indicating that incorporating restful sleep, effective stress management, and nurturing positive social connections are essential for promoting and sustaining optimal health [16]. However, the COPD guidelines primarily address specific aspects of lifestyle, such as smoking and physical activity, while overlooking mental health, alcohol consumption, sleep, and social connections [17]. Previous studies that accounted for the above lifestyle factors have shown that unhealthy lifestyle behaviors could be associated with progression and worse outcomes of COPD [18,19,20,21,22]. Nonetheless, most of these studies have focused on individual lifestyle factors rather than examining the comprehensive impact of multiple behaviors on COPD outcomes. Furthermore, determining the level to which patients with COPD adopt these healthier lifestyle behaviors among patients with COPD is challenging, as it varies worldwide and is influenced by factors such as socioeconomic status and access to healthcare [23,24,25,26,27].

While the importance of the four main lifestyle risk factors in patients with COPD has been emphasized, there is still a lack of empirical evidence on the inclusion of sufficient sleep and its impact on the quality of life in patients with COPD. More specifically for Greece, the level of adherence among patients with COPD to the recommended lifestyle changes has not been adequately explored [28,29,30]. Given the high prevalence of COPD in Greece [31,32], there is an urgent need to address the aforementioned knowledge gaps. Consequently, the present study had two objectives: (a) to describe and analyze the prevalence of five lifestyle factors (smoking, physical activity, alcohol consumption, body mass index, and sleep duration), and (b) to assess the association between the degree of clustering of lifestyle behaviors and the quality of life among patients with COPD in primary care settings in Greece.

## 2. Materials and Methods

### 2.1. Design and Sample

This cross-sectional study included 253 patients with COPD who were over 40 years old from the prospective COCARE COPD study recruited from six primary care centers in Crete, Greece. The participants were invited to participate and were given information about the study objectives during their scheduled visit to a primary care center. If they agreed to participate, they were asked to provide written consent, and then anonymously completed the questionnaires. This study had the following inclusion criteria, which patients had to meet: (a) >40 years, (b) have a physician-diagnosed COPD confirmed with spirometry, and (c) have an educational background beyond elementary school. On the other hand, we applied the following criteria to exclude certain individuals: patients with severe neurological or mental disorders, pregnancy, those who had recently experienced a COPD exacerbation, individuals with limited understanding of the Greek language, and those who declined to participate.

### 2.2. Data Collection

A comprehensive evaluation of the participants was conducted, which assessed various demographic parameters, such as age, gender, educational level, body mass index (BMI), physical activity, smoking status, alcohol consumption, comorbidities, and exacerbation status using a self-reported questionnaire (created for this study). In addition, COPD-specific quality of life was assessed using the COPD assessment test (CAT).

### 2.3. Study Tools

#### 2.3.1. The Healthy Lifestyle Index

After a thorough review of the literature, the researchers created the healthy lifestyle index (HLI), which is based on five modifiable lifestyle factors: active smoking, leisure-time physical activity, alcohol consumption, BMI, and sleep duration. Each lifestyle factor was dichotomized as healthy or unhealthy and each factor was then assigned a score of 0 and 1 for unhealthy and healthy, respectively. Healthy criteria for lifestyle factors were no active smoking, being physically active (150 min/week or more) [33], moderate alcohol consumption (14 units/week or less) [34], having a healthy weight (BMI less than 30 kg/m^2^), and adequate sleep (7–9 h per night) [35]. HLI score was defined as the sum of these five lifestyle factors scores; thus, the HLI ranged from zero (least healthy) to five (healthiest) points. These scores were then categorized into two levels of health: optimal (3–5 points) and poor (0–2 point).

#### 2.3.2. COPD Assessment Test (CAT) Questionnaire

The COPD assessment test (CAT) is a straightforward questionnaire used to evaluate the self-reported impact of COPD on an individual’s health status [36]. It has been translated and validated in Greek [36]. Moreover, CAT is comprises eight items, including cough, phlegm, chest tightness, breathlessness, limited activities, confidence in leaving home, sleeplessness, and energy; patients rate their experience on a scale of 0 to 5 [37]. The total score ranges from 0 to 40, with higher values indicating a poorer health status. Furthermore, a cutoff point of 10 or higher is utilized to identify the presence of poor health status. The minimal clinically important difference (MCID) for CAT is a change of 2 points [38].

### 2.4. Statistical Analysis

The Shapiro–Wilk test was used to examine the normality. The continuous variables with normal distribution are presented by mean and standard deviation (SD), and the continuous variables with non-normal distribution are presented by median and 25th–75th percentile. The categorical variables are expressed as frequency and percentage. Comparisons between groups were evaluated by *t*-test (normally distributed continuous variables) and Mann–Whitney U-test (not normally distributed continuous variables). Further-more, the chi-square test was employed for categorical variables. Logistic regression was used to estimate adjusted odds ratios (ORs) and 95% confidence intervals (CIs) to examine the associations between each individual healthy lifestyle factor and the overall impact of healthy lifestyle on CAT score. CAT score was dichotomized according to predetermined, clinically relevant cutoff values. CAT score was also evaluated as a continuous variable using linear regression, as CAT followed a normal distribution. Effect estimates for linear regression are presented in terms of beta coefficient and 95% CIs. All models were adjusted for age, sex, education, marital status, and comorbidities. Age was considered continuously and categorically as age groups of 40–59, 60–69, and ≥70 years; BMI was also considered continuously and categorically as BMI groups of <30 and ≥30 kg/m^2^. Two-sided *p*-values were computed and a *p*-value < 0.05 was considered significant. All analyses were performed with SPSS software (version 25, SPSS Inc., Chicago, IL, USA).

## 3. Results

A total of 253 patients with COPD participated in this study; however, 17 patients (7%) were excluded from the present analysis because of missing data for at least one component of the HLI. This resulted in a final sample size of 236 participants. The mean age of the patients included in this study was 68 years (range 43–90 years). The patients were mostly male (67%) and married (76%). Almost half of the participants had completed primary education (49%), whereas 36% and 15%, respectively, had a secondary and higher level of education (Table 1).

**Figure 1:**
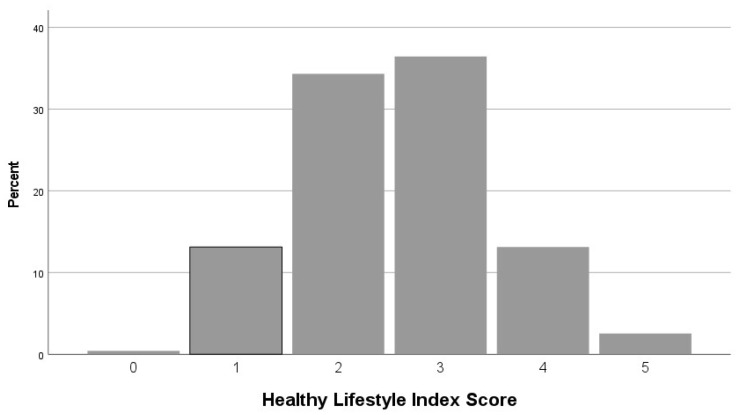
Prevalence of engaging in health-related behaviors among patients with COPD.

With regard to the HLI index components, the most prevalent healthy behavior was nondrinking or moderate drinking (92%), followed by non-obesity (56%, BMI < 30 kg/m^2^), current nonsmoking (50%), exercise for at least 150 min/week (32%), and adequate sleep (25%). The HLI mean value was 2.6 (SD 0.9), indicating a moderately healthy lifestyle in our population (possible range of the HLI is 0–5, with higher values corresponding to healthier lifestyle). A very small proportion of respondents (1 patient, 0.4%) refrained from engaging in any of the five healthy behaviors; 31 (13%) engaged in one behavior; 81 (34%) in two behaviors; 86 (36%) in three behaviors; and 31 (13%) in four behaviors; only 6 (3%) reported engaging in all five behaviors (Figure 1). An optimal lifestyle score (at least three out of five healthy behaviors) was observed in 52% of the patients. The HLI distribution was used to divide the studied population into two groups: one with optimal scores (ranging from 3 to 5 points) and another with poor scores (ranging from 0 to 2 points).

According to the CAT score (range 0–29), 155 (66%) patients were categorized as medium or high impact of COPD on the patient’s life (CAT score ≥ 10) with a mean CAT score of 12.3 (SD = 6.0). More specifically, the total number of patients with CAT scores < 10, 10–20, and >20 was 81 (34%, low impact), 131 (56%, medium impact), and 24 (10%, high impact), respectively. Moreover, 34 (14%) patients had experienced at least two exacerbations during the last year. Table 2 summarizes the baseline healthy lifestyle factors and the CAT score of the 236 participants categorized into poor (48%) and optimal (52%) lifestyle groups. There were no notable differences between the groups, except for a slightly higher average age in the optimal group compared to the poor lifestyle group (69 vs. 67, *p* = 0.02). As expected, patients in the optimal lifestyle group compared to poor lifestyle group showed healthier behaviors, including higher prevalence of regular physical activity (55 vs. 7%, *p* < 0.001) and adequate sleep (39 vs. 11%, *p* < 0.001). They were also less likely to smoke (29 vs. 72%, *p* < 0.001) or have high alcohol consumption (2 vs. 13%, *p* < 0.001). Furthermore, the optimal lifestyle group had a lower prevalence of obesity (27 vs. 62%, *p* < 0.001). Regarding comorbidities, dyslipidemia and hypertension were the most prevalent comorbidities, with 59% and 55% of participants being affected, respectively. This was followed by diabetes type 2 (31%), coronary artery disease (CAD) (23%), asthma (17%), gastroesophageal reflux (14%), obstructive sleep apnea (14%), osteoporosis (13%), depression (13%), cancer (11%), and atrial fibrillation (9%). Less common conditions included rheumatic disease, heart failure, other types of arrhythmia, anxiety disorder, cerebrovascular accidents, and transient ischemic attacks, each affecting less than 7% of the participants. The prevalence of these conditions varied to some extent between the poor HLI and optimal HLI groups, but no statistically significant differences were observed between the groups (*p* < 0.005).

### 3.1. Healthy Lifestyle Behaviors and COPD-Specific Quality of Life

Higher HLI scores were associated with decreased CAT score after adjustments for age, gender, education, marital status, and comorbidities. More specifically, every 1-point increment of HLI was associated with a decrease of 2.3 points in the CAT score (95% CI −3.1 to −1.5, *p* < 0.001) and optimal HLI (3 or higher) was positively associated with a decrease of 3.6 points in the CAT score (95% CI −5.1 to −2.0).

The results were similar when we considered the dichotomized CAT score (*p*-trend <  0.001) (Table 3). Higher HLI values were negatively associated with the odds of being categorized as medium or high impact of COPD on the patient’s life (CAT score ≥ 10). Specifically, in the unadjusted model, each one-point increase in the healthy lifestyle score was linked to 51% lower odds of being categorized as medium or high impact (CAT ≥ 10) (OR: 0.49, 95% CI: 0.358–0.669, *p* < 0.001). This inverse relationship remained robust after adjusting for demographic and socioeconomic factors (OR: 0.433, 95% CI: 0.303–0.619, *p* < 0.001) (Model I) and persisted even with additional adjustments for comorbid conditions such as diabetes, hypertension, and cardiovascular diseases (OR: 0.424, 95% CI: 0.294–0.612, *p* < 0.001) (Model II). When categorizing individuals by their HLI, those with optimal HLI score had significantly reduced odds of being categorized as medium or high impact compared to those with poor HLI. Specifically, patients with optimal lifestyle behaviors exhibited a striking reduction in the odds of being categorized as medium or high impact, up to 69% in the adjusted Model I (OR: 0.317, 95% CI: 0.172–0.582, *p* < 0.001), with similar reductions observed in Model II (OR: 0.310, 95% CI: 0.167–0.575, *p* < 0.001).

**Figure 2:**
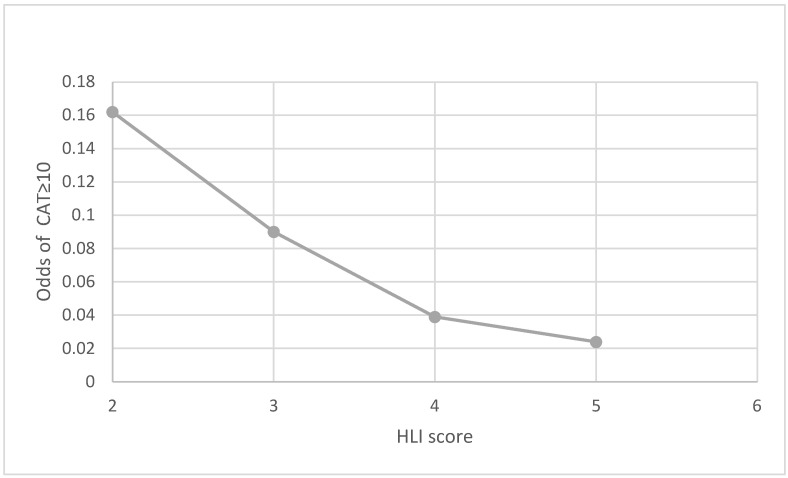
The linear relationship between healthy lifestyle score and the odds of being categorized as medium or high impact of COPD on the patient’s life (CAT score ≥ 10). Given the limited number of subjects without any lifestyle behaviors, the 0 and 1 categories in the healthy lifestyle index (HLI) score were merged together into a single category represented by “1”. The models were conducted after adjusting by age, sex, education, marital status, diabetes type 2, hypertension, hyperlipidemia, cardiovascular diseases, asthma, and obstructive sleep apnea. Odds ratios for each HLI score were estimated using “1” as the reference category.

This relationship is further illustrated in Figure 2, which depicts the linear association between the healthy lifestyle score and the odds of being categorized as medium or high impact, highlighting the reduced likelihood of poor health status with higher lifestyle scores.

### 3.2. Subgroup and Separate Analyses

Table 4 presents the separate analyses focusing on the association between individual healthy lifestyle factors and COPD health status after adjustments for confounders. Non-/former smoking was significantly associated with reduced odds of being categorized as medium or high impact (CAT ≥ 10) (OR: 0.543, 95% CI: 0.282–1.049, *p* = 0.039). Adequate sleep also showed a significant association with lower likelihood of being categorized as medium or high impact (OR: 0.337, 95% CI: 0.160–0.710, *p* = 0.004). Increased PA (at least 150 min/week) showed the strongest protective effect against worse health status (OR: 0.238, 95% CI: 0.122–0.463). However, low consumption of alcohol and low BMI did not show a significant association with COPD health status after adjusting for multiple confounders and other lifestyle factors.

Table 5 presents the association between healthy HLI score and being categorized as medium or high impact (CAT ≥ 10) stratified by subgroup. Across different demographic groups, high healthy lifestyle score was consistently associated with lower odds of being categorized as medium or high impact. Stratified by age, the 40–59 age group exhibited the most significant reduction, being categorized as medium or high impact with higher HLI score (OR 0.051, 95% CI: 0.006 to 0.426). Males showed a slightly stronger protective effect compared to females (OR: 0.438, 95% CI: 0.238–0.678). Between different areas of residence, the most substantial reduction in high HLI score on the risk of being categorized as having poor COPD health status was observed in the rural group (OR: 0.327, 95% CI: 0.179–0.597). Higher education levels were associated with a greater reduction of being categorized as medium or high impact with high HLI index score (OR: 0.171, 95% CI: 0.037–0.796).

## 4. Discussion

This study examined the lifestyle behaviors of patients with COPD in primary care settings in Greece and their potential combined effect on their disease-specific quality of life. Our findings indicate that a significant proportion (48%) of the participants failed to adhere to a minimum of three out of five healthy behaviors. Additionally, a higher number of healthy lifestyle factors defined by a high HLI score was independently associated with a better disease-specific quality of life, adjusted for age, gender, area of residence, and education level. However, it should be noted that this association was more pronounced among younger participants, those with higher levels of education, and those residing in rural areas.

Patients with COPD, despite being aware of their chronic respiratory disease and perceiving their health as worse than the general population [39,40], continue to engage in unhealthy lifestyle behaviors. The results of our analysis showed that the prevalence of healthy behaviors was less than optimal, especially for certain lifestyle factors. More specifically, half of the participants indicated that they currently smoke, which is a significant percentage compared to the wide percentage of current smokers (ranging from 1.4% to 47%) found in previous studies in patients with COPD [41,42,43,44]. The significant prevalence of this addictive behavior, which is also considered as a chronic relapsing disease [45], must be carefully considered due to its substantial impact on the health status of patients with COPD. Our study, as well as previous studies [21,46,47,48], consistently show that nonsmokers and former smokers experience significantly better health status compared to current smokers. This knowledge holds great significance as a source of motivation for general practitioners (GPs) and other healthcare professionals to provide more intense smoking cessation support to patients with COPD [49]. Moreover, it is crucial to acknowledge that most patients with COPD (68%) in our study did not make regular physical activity a part of their routine, which is in line with previous studies [50,51,52]. A potential explanation for this finding could be that patients with COPD often experience dyspnea-related kinesiophobia, resulting in physical inactivity [53]. Furthermore, the high impact of COPD on a patient’s life associated with physical inactivity found in our study and other research [54,55,56] provides strong support for the implementation of organized physical activity programs in primary care settings for patients with COPD.

The most concerning prevalence of healthy behavior in our population was that only 25% reported getting enough sleep. Ensuring an appropriate duration of sleep is vital for promoting optimal health [57]. While it is widely acknowledged that poor sleep quality significantly impacts the health and overall well-being of patients with COPD [58,59,60], limited research has been conducted on the correlation between sleep duration and health status in COPD patients [61]. In the present study, adequate sleep was significantly associated with a 66% reduced likelihood of having poor health status. Therefore, our results suggest that sleep duration might be a modifiable risk factor for health status in patients with COPD. Interestingly, CAT is the only tool/questionnaire among those commonly used for assessing COPD that includes one item which evaluates sleep [36]. This highlights the need for more tools/questionnaires to incorporate sleep assessment for the evaluation of the overall COPD health status. However, more studies are required to explore whether interventions that promote adequate sleep duration could improve the quality of life in this population.

Another important treatable lifestyle behavior affecting COPD to consider is alcohol consumption. Previous research has suggested that low to moderate alcohol consumption may lead to a reduced risk of symptom burden in patients with COPD [62]. In our study, most of the population (92%) reported nondrinking or moderate drinking, a percentage that is higher than that reported in previous studies [63,64,65,66]. Research exploring the role of nondrinking or moderate drinking on COPD health status is limited to one study, which suggests that alcohol consumption does not change the quality of life or depressive mood in patients with obstructive lung diseases [67].

To the best of our knowledge, our study is the first to explore the association between a combination of healthy lifestyle factors and disease-specific quality of life in patients with COPD. Moreover, a strong correlation was noted between COPD-specific quality of life and the clustering of five modifiable lifestyle factors—smoking, physical activity, alcohol consumption, BMI, and adequate sleep—into the HLI. Specifically, each one-point increase in the HLI was associated with a decrease in CAT score by 2.3 points, and with 51% lower odds of having poor COPD health status, indicating an improvement in disease-specific quality of life. Moreover, our results suggest that a high HLI score was positively associated with improved health status, independent of age, gender, area of residence, or education level. Limited research exists regarding the association between the combinations of healthy behaviors, as measured by the HLI, and health outcomes in patients with COPD, and none of these studies have accounted for sleep as one of the factors of a healthy lifestyle or assessed disease-specific quality of life [65,68]. Therefore, our findings underscore the potential benefits of a comprehensive approach to lifestyle management for these patients.

The results of our study have significant implications for both clinical practice and public health. Individual counseling by clinicians and national-level public policies to decrease smoking, alcohol consumption, and body weight; increase exercise frequency; and ensure adequate sleep are essential to reduce the burden of COPD. By investing extra time in motivating, counseling, and coaching patients for adapting to a healthy lifestyle, medical practitioners can play a crucial role in reducing the burden of COPD. Therefore, there is an urgent need to enhance the learning and expertise of practicing physicians, especially primary care practitioners, in the field of lifestyle medicine. Additionally, policymakers could implement educational programs to equip healthcare professionals with the necessary knowledge and skills to more effectively promote these behaviors in their patients. Ultimately, these initiatives could encourage healthier habits and reduce the global burden of COPD.

### Limitations

Despite its strengths, our study has a few limitations worth mentioning. First, since the patients were specifically recruited from southern Greece, we cannot generalize our findings to the entire population of Greece. Second, the cross-sectional design precludes the establishment of causality; we can only infer associations between lifestyle behaviors and health outcomes. Longitudinal studies are needed to confirm these findings and to explore the causal pathways linking lifestyle modifications to improved quality of life in patients with COPD. Third, self-reported data on lifestyle behaviors and health status may be subject to recall bias and social desirability bias, potentially influencing the accuracy of our measurements. Finally, factors, such as socioeconomic status and dietary habits, that could influence both lifestyle behaviors and health outcomes, were not fully accounted for in our analysis. Nonetheless, we do not anticipate any major variations in dietary habits, as our study only included subjects from Crete who live in the same area and have similar dietary habits. Future research could aim to include a broader range of confounding variables to better isolate the effects of lifestyle behaviors on COPD health status.

## 5. Conclusions

In conclusion, the findings from our study indicate that a large proportion of patients with COPD did not adopt healthy behaviors, specifically in relation to smoking, exercise, normal BMI, and adequate sleep. Additionally, our findings suggest that by following a healthy lifestyle, patients with COPD could improve their quality of life. Therefore, primary healthcare professionals could utilize behavioral interventions in the overall management of COPD to improve the quality of life of their patients. Furthermore, healthcare providers could significantly improve the management of COPD and patient outcomes by targeting and improving these lifestyle behaviors with comprehensive intervention strategies.

## Figures and Tables

**Table 1:** Demographic characteristics of the 236 participants according to their HLI score.

Demographic Characteristics	Overall N = 236	Poor HLI Group (HLI = 2 or Lower) N = 113	Optimal HLI Group (HLI = 3 or Higher) N = 123	*p*-Value
Age (years)	68 ± 9	67 ± 8	69 ± 9	0.029
Age group ≥ 60 years	191 (81)	87 (77)	104 (85)	0.140
**Sex**				0.462
Male	158 (67)	73 (65)	85 (69)	
Female	78 (33)	40 (35)	38 (1)	
**Area of residence**				0.423
Rural	94 (40)	42 (37)	52 (42)	
Urban	142 (60)	71 (63)	71 (58)	
**Education level**				0.333
Primary level	112 (47)	50 (45)	62 (50)	
Secondary level	90 (38)	49 (43)	41 (33)	
Higher level	34 (15)	14 (12)	20 (17)	
**Married**				0.292
No	42 (18)	23 (20)	19 (15)	
Yes	194 (82)	90 (80)	104 (85)	

Data are presented as N (%) for categorical variables and mean values ± SD or median (25th–75th percentile) for continuous variables. A two-tailed *t*-test for independent samples was used for continuous variables and the chi-square test was used for categorical variables. *p* < 0.05 was considered to indicate a statistically significant difference. HLI: healthy lifestyle index.

**Table 2:** Basic characteristics of the 236 participants according to their HLI score.

Healthy Lifestyle Factors	Overall N = 236	Poor HLI Group (HLI = 2 or Lower) N = 113	Optimal HLI Group (HLI = 3 or Higher) N = 123	*p*-Value
**Smoking**				
Non-/former smokers	119 (50)	32 (28)	87 (71)	<0.001
**Physical activity**				
At least 150 min/week	75 (32)	8 (7)	67 (55)	<0.001
**Alcohol consumption**				
Less than 14 units/week	218 (92)	98 (87)	120 (98)	0.002
**BMI**				
Less than 30 kg/m^2^	133 (56)	43 (38)	90 (73)	<0.001
**Sleep**				
Adequate sleep (7–9 h)	60 (25%)	12 (11%)	48 (39%)	
**Healthy lifestyle index**	2.6 (0.9)	1.7 ± 0.5	3.3 ±0.6	<0.001
**Clinical characteristics**				
CAT score	12.4 ±5.9	14.2 ± 5.9	10.7 ±5.5	<0.001
CAT score ≥ 10	155 (66)	89 (79)	66 (54)	<0.001
Exacerbations ≥ 2	34 (14)	19 (17)	15 (12)	0.313
**Comorbidities**				
Hypertension	130 (55)	64 (57)	66 (54)	0.646
Coronary artery disease (CAD)	53 (23)	22 (20)	31 (25)	0.292
Atrial Fibrillation	20 (9)	8 (7)	12 (10)	0.461
Other type of arrhythmia	13 (6)	7 (6)	6 (5)	0.658
Heart failure	13 (6)	8 7)	5 (4)	0.311
Cerebrovascular accident	5 (2)	3 (3)	2 (2)	0.583
Transient ischemic attack	2 (1)	1 (1)	1 (1)	0.952
Gastroesophageal reflux	34 (14)	18 (16)	16 (13)	0.523
Cancer	26 (11)	12 (11)	14 (11)	0.980
Diabetes type 2	73 (31)	41 (36)	32 (26)	0.088
Dyslipidemia	138 (59)	66 (58)	72 (59)	0.984
Osteoporosis	31 (13)	13 (12)	18 (15)	0.477
Asthma	41 (17)	20 (18)	21 (17)	0.945
Anxiety Disorder	14 (6)	7 (6)	7 (6)	0.870
Depression	30 (13)	18 (16)	12 (10)	0.155
Obstructive Sleep apnea	32 (14)	17 (15)	15 (12)	0.523
Rheumatic disease	16 (7)	11 (10)	5 (4)	0.084

Data are presented as N (%) for categorical variables and mean values ± SD or median (25th–75th percentile) for continuous variables. A two-tailed *t*-test for independent samples was used for continuous variables and the chi-square test was used for categorical variables. *p* < 0.05 was considered to indicate a statistically significant difference. HLI: healthy lifestyle index.

**Table 3:** Association between healthy lifestyle index and COPD-specific quality of life (CAT score ≥ 10).

	Non-Adjusted OR (95% CI) *p*-Value	Model I OR (95% CI) *p*-Value	Model II OR (95% CI) *p*-Value
Healthy lifestyle index score	0.490 (0.358–0.669), *p* < 0.001	0.433 (0.303–0.619), *p* < 0.001	0.424 (0.294–0.612), *p* < 0.001
HLI = 2 or lower	Ref	Ref	Ref
HLI = 3 or higher	0.312 (0.176–0.554), *p* < 0.001	0.317 (0.172–0.582), *p* < 0.001	0.310 (0.167–0.575), *p* < 0.001

Abbreviations: 95% CI: 95% confidence interval; HLI: healthy lifestyle index. Non-adjusted model: adjusted for no variables. Model I adjusted for age, sex, education, and marital status. Model II adjusted for age, sex, education, marital status, diabetes type 2, hypertension, hyperlipidemia, cardiovascular diseases, asthma, and obstructive sleep apnea.

**Table 4:** Separate analyses on the associations between COPD health status (CAT score ≥ 10) and healthy lifestyle factors.

Healthy Lifestyle Factors	OR (95% CI)	*p*-Value
Non-/former smoking	0.543 (0.282, 1.049)	0.039
Active physical activity (at least 150 min/week)	0.238 (0.122, 0.463)	<0.001
Alcohol less than 14 units/week	0.827 (0.239, 2.860)	0.764
BMI less than 30 kg/m^2^	0.664 (0.327, 1.349)	0.257
Adequate sleep (7–9 h)	0.337 (0.160–0.710	0.004

Abbreviations: 95% CI: 95% confidence interval. Adjusted for age, sex, education, marital status, diabetes type 2, hypertension, hyperlipidemia, cardiovascular diseases, asthma, and obstructive sleep apnea. The healthy lifestyle behaviors were also adjusted for each other: non-/former smoking, active physical activity, moderate or less drinking, non-obesity, and adequate sleep.

**Table 5:** Associations between COPD health status and HLI score across different demographic groups.

Demographic Characteristics	OR (95% CI)	*p*-Value
**Age**		
Age group 40–59 years	0.051 (0.006–0.426)	0.006
Age group 60–69 years	0.476 (0.268–0.846)	0.011
Age group ≥ 70 years	0.495 (0.274–0,895)	0.020
**Sex**		
Male	0.438 (0.283–0.678)	<0.001
Female	0.440 (0.232–0.835)	0.012
*p*-value	0.821	
**Area of residence**		
Rural	0.327 (0.179–0.597)	<0.001
Urban	0.500 (0.312–0.801)	0.004
**Education level**		
Primary level	0.404 (0.235–0.695)	0.001
Secondary level	0.532 (0.312–0.908)	0.021
Higher level	0.171 (0.037–0.796)	0.024

## Data Availability

The data that support the findings of this study are available from the corresponding author upon reasonable request, due to privacy restrictions.
